# Turning Biodiesel Waste Glycerol into 1,3-Propanediol: Catalytic Performance of Sulphuric acid-Activated Montmorillonite Supported Platinum Catalysts in Glycerol Hydrogenolysis

**DOI:** 10.1038/s41598-018-25787-w

**Published:** 2018-05-10

**Authors:** Shanthi Priya Samudrala, Shalini Kandasamy, Sankar Bhattacharya

**Affiliations:** Department of Chemical Engineering, Monash University, Melbourne, 3800 Australia

## Abstract

Direct C-O hydrogenolysis of bioglycerine to produce 1,3-propanediol selectively is a vital technology that can expand the scope of biodiesel industry and green chemical production from biomass. Herein we report sulphuric acid-activated montmorillonite clay supported platinum nanoparticles as highly effective solid acid catalysts for the selective production of 1,3-propanediol from glycerol. The catalytic performances of the catalysts were investigated in the hydrogenolysis of glycerol with a fixed bed reactor under ambient pressure. The results were found promising and showed that the activation of montmorillonite by sulphuric acid incorporated Brønsted acidity in the catalyst and significantly improved the selectivity to 1,3-propanediol. The catalytic performance of different platinum loaded catalysts was examined and 2 wt% Pt/S-MMT catalyst presented superior activity among others validating 62% 1,3-propanediol selectivity at 94% glycerol conversion. The catalytic activity of 2Pt/S-MMT was systematically investigated under varying reaction parameters including reaction temperature, hydrogen flow rate, glycerol concentration, weight hourly space velocity, and contact time to derive the optimum conditions for the reaction. The catalyst stability, reusability and structure-activity correlation were also elucidated. The high performance of the catalyst could be ascribed to well disperse Pt nanoparticles immobilized on acid-activated montmorillonite, wider pore-structure and appropriate acid sites of the catalyst.

## Introduction

Sustainable fuel and chemical production by utilization of biomass-derived feedstocks is a very attractive approach compared to conventional petroleum-based fuel and chemical production. Petroleum-based fuel causes serious ecological damage by greenhouse gas emissions and eventually depletes the world’s natural resources^1^. Biodiesel is one such renewable, eco-friendly fuel produced from biomass, that can directly replace conventional petroleum diesel, and has attained great industrial expansion world-wide. For every ten quintals of biodiesel synthesized by base catalyzed transesterification of plant triglycerides, about one quintal of waste glycerol is coproduced, nearly 10 wt% of the complete product (Supplementary Fig. S1). With the rising biodiesel production, its major co-product, crude glycerol, is set to reach a global production of six million tons by 2025^2^. The large volume of accumulated glycerol devalues the industrial biodiesel production process, dropping the market price of glycerol. Moreover, discarding surplus glycerol involves costly procedure and is one of the main difficulties provoked by biodiesel industries. Hence, there is an urgent need to manage the glycerol by-product in biodiesel production. This includes finding new applications for glycerol such as chemical production, or energy generation, to improve the long-term viability of the biodiesel production industry.

Due to the biodegradable, non-toxic as well as highly functionalized nature, glycerol is considered one of the top twelve bio-based platform chemicals by US Department of Energy^3^. Significant global interest has been focused on the utilization of biodiesel waste glycerol as a versatile building block and its transformation into high value-added specialty chemicals. Heterogeneous catalysis is an important approach to establish sustainable chemical reactions and develop new methods for glycerol valorization. Several catalytic processes including hydrogenolysis, oxidation, dehydration, aqueous phase reforming, esterification, and acetalization have been established directing at upgrading glycerol to value-added chemicals and fuels^4–7^. Amongst various processes, hydrogenolysis of glycerol to propanediols (1,2-propanediol & 1,3-propanediol) is a promising approach for value-added chemical production. 1,3-propanediol (1,3-PDO) is an important industrial commodity and a building block for wide-ranging products including polymers, paints, cosmetics, cleaning products, adhesives, carpets, textiles, coolant, and personal care. The foremost application of 1,3-PDO is the manufacture of polytrimethylene terephthalate (PTT), a commercial polyester used to produce carpet fibers. The expanding use of 1,3-PDO creates high demand for the product, and the global market value is expected to reach USD 776.3 million by 2022^8^. Conventionally, 1,3-PDO is produced by petrochemical methods involving hydration of acrolein or hydroformylation of ethylene oxide followed by hydrogenation^9^. Owing to the commercial importance and rising consumption of 1,3-PDO in various end-use industries, there is a necessity to produce 1,3-propanediol via sustainable and economically viable processes. 1,3-PDO produced directly from bio-glycerol catalytic hydrogenolysis will provide a sustainable route with a greater emphasis in current chemical processes.

**Figure 1:**
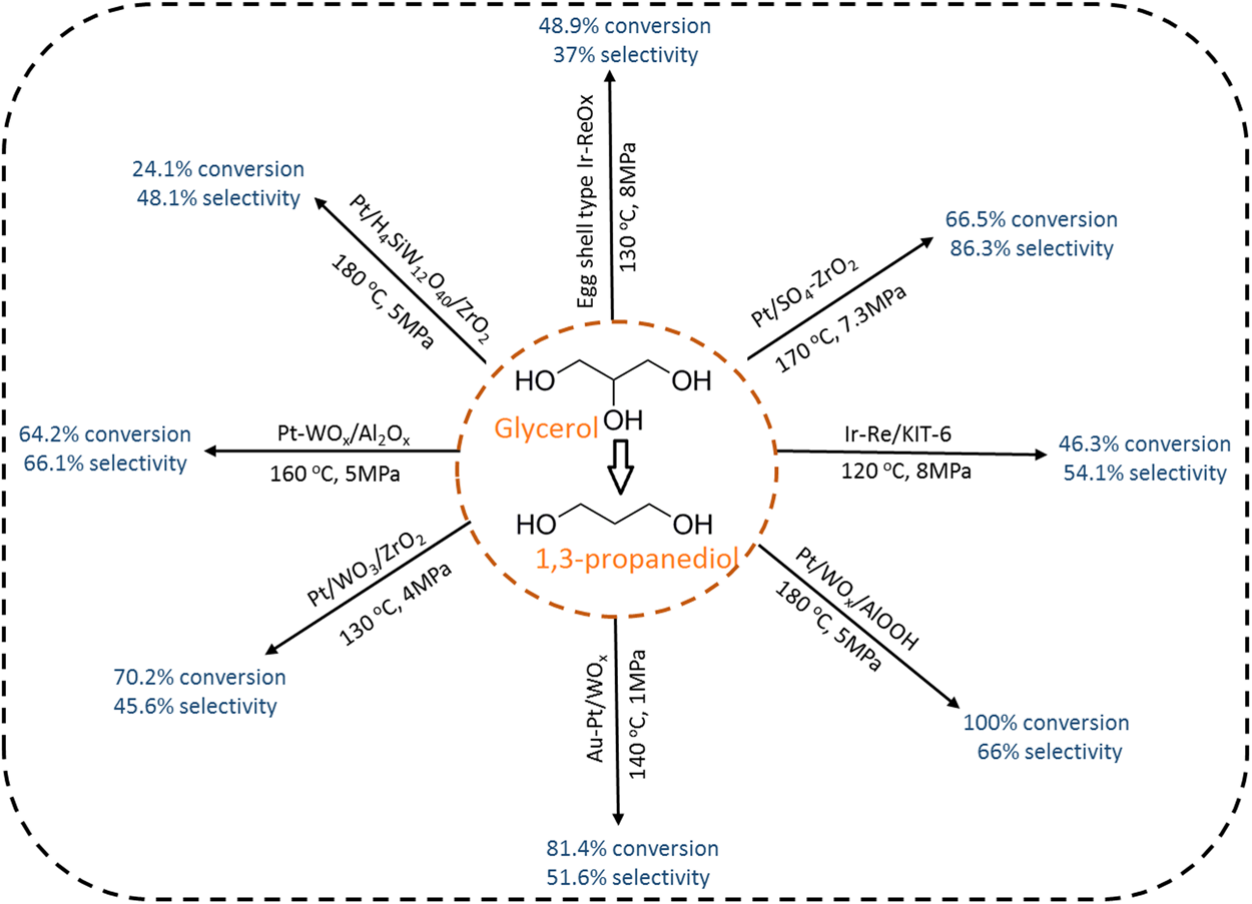
Catalysts investigated for glycerol hydrogenolysis to 1,3-PDO in literature.

The catalytic reaction of glycerol hydrogenolysis favoring 1,2-PDO by now is well established and has been demonstrated to be a relatively feasible process. However, selective production of 1,3-PDO which involves the removal of sterically hindered secondary hydroxyl group from glycerol has turned out to be far more intricate than that of 1,2-PDO, and the reported systems are limited. Supported noble metal (Pt, Ir) catalysts with a co-catalyst (WO_3_, ReOx) especially high in Brønsted acid sites have been proven to be the more efficient catalytic materials for the selective production of 1,3-PDO from glycerol hydrogenolysis^10–17^ (Fig. 1). Although some attempts^11,17^ for innovative catalysts favoring 1,3-PDO have been made by researchers, the quest for new highly active and selective catalysts for 1,3-PDO from glycerol hydrogenolysis has been a longstanding challenge. Hence, with a fundamental understanding of the mechanism of glycerol hydrogenolysis reaction, there is a need to design better and more efficient catalysts with high selectivity.

Montmorillonite K10 (MMT) is a non-corrosive, low-cost, environmentally benign solid acid catalyst that can exist in both normal and ion-exchanged forms. MMT has been extensively explored as a highly active catalyst and a support material for various chemical transformations and possess unique properties such as, easy handling, recovery and probable tuning of acid sites^18^. The interlayer space of MMT provides a good platform to accommodate various guest species or metal nanoparticles^19^. The modification of montmorillonite by acid treatment can significantly increase the acid catalytic performance of montmorillonite. Its combination with noble metal Platinum which has been demonstrated to hold high hydrogenation activity and selectivity in C–O bond hydrogenolysis^20–22^, will therefore be a prospective promising formulation to obtain high yields of 1,3-PDO.

To achieve an improved catalytic activity and selectivity to 1,3-PDO in hydrogenolysis of glycerol, the present work focused on the development of highly efficient, eco-friendly and recyclable sulfuric acid-activated MMT supported platinum nanoparticles as a potential catalyst promoting selective production of 1,3-PDO. Different loadings of platinum nanoparticles immobilized on MMT catalysts were prepared, thoroughly characterized by different characterization techniques and evaluated in vapor phase glycerol hydrogenolysis using a fixed-bed reactor under mild reaction conditions (moderate temperature and atmospheric pressure). The effect of process variables on the catalytic performance was carefully investigated. The catalyst stability and reusability has also been evaluated. The research work reported herein is unique and to the best of our understanding, platinum supported on sulfuric acid-activated montmorillonite has not been explored so far as a catalyst for glycerol conversion to 1,3-PDO by hydrogenolysis.

## Results

### Catalyst characterization

#### Powder X-ray Diffraction analysis

**Figure 2:**
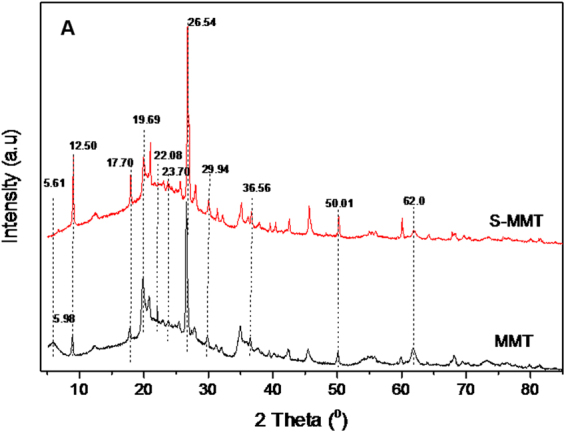
XRD patterns of MMT and S-MMT catalysts.

The diffraction profiles of montmorillonite, sulfuric acid-treated montmorillonite and various loadings (0.5–3 wt%) of Pt/S-MMT catalysts are shown in Figs 2 and S2 (Supplementary). The XRD pattern of parent montmorillonite (MMT) (Fig. 2) showed a characteristic (001) reflection of layered structure at approximately 2θ of 5.98°. The (001) reflection of sulfuric acid-treated montmorillonite (S-MMT) retained with a slight shift to 2θ of 5.61° and appeared less intense than that of parent MMT^23^.

The other characteristic reflections at 2θ 12.5°, 17.7°, 19.7°, 22.08°, 23.7°, 26.54°, 29.94°, 36.56°, 50.01°, 62.0° assigned to the crystal lattice planes of d 060 (0.502 nm), 080 (0.451 nm), 020 (0.378 nm), 060 (0.303 nm), 030 (0.171 nm), 050 (0.151 nm) and 050 (0.148 nm) respectively^24^, remained clearly identified in both MMT and S-MMT. However, a significant increase in the intensity of peaks at 2θ 12.5°, 17.7° & 26.54° was noted in the XRD pattern of sulfuric acid-activated montmorillonite. The persistence of all the above diffraction peaks in S-MMT clearly documents that the parent MMT crystalline structure was intact and sulfuric acid treatment did not considerably alter the layered structure of montmorillonite.

For the samples of 0.5Pt/S-MMT, 1Pt/S-MMT, 2Pt/S-MMT & 3Pt/S-MMT, all the crystal lattice planes appeared almost unchanged (Supplementary Fig. S2). The intercalation of Pt metal nanoparticles onto the layered structure of MMT material showed no impact on its crystallinity, however, significant increase in peak intensity was observed at 2θ 17.7° & 26.54° as the Pt loading was increased. The characteristic peaks corresponding to the formation of metallic Pt (0) phase that generally appears at 2θ 40.5°, 47.0°, and 68.6°, allocated to (111), (200), and (220) reflections of face-centered cubic (fcc) platinum lattice, were not seen in the XRD pattern (12). This could be due to the high dispersion of Pt on surface of MMT and the crystallite size may possibly be very low (<4 nm), which is below the detection limit of XRD. Thus, XRD results infer that the general crystalline structure of montmorillonite was well retained with no change in d-spacing and intercalation structure, even after the sulfuric acid treatment and the introduction of Pt particles into the inter lamellar structure of montmorillonite.

#### Fourier transform-infrared spectral analysis

The FTIR spectra of parent MMT, S-MMT and 0.5–3 wt% Pt incorporated samples are displayed in Fig. S3 (Supplementary). All the samples exhibited the absorbance bands representative of montmorillonite^25^. The absorbance bands at 526 cm^−1^ and 462 cm^−1^ were attributed to the Al-O stretching and Si-O bending vibrations of Si-O-Al groups, respectively. While those peaks around 818 cm^−1^ and 916 cm^−1^ corresponds to Mg-OH-Al and Al-O-Al bending vibrations respectively, the bands at about 1050 cm^−1^ and 1164 cm^−1^ were attributed to Si-O stretching vibration. The OH vibrations arising from hydrated cations in the interlayer appeared at about 1474 and 1378 cm^−1^. The absorbance bands at 3648 cm^−1^ and 1638 cm^−1^ resulted from –OH stretching and bending vibrations respectively. Therefore, the acid activation and Pt incorporation did not destruct the layered structure of montmorillonite and no significant change in the structure, as evident from FTIR spectra.

#### BET surface area and pore volumes

As summarized in Table S1 (Supplementary), the specific surface area of MMT catalyst calculated from BET equation was found to be 188 m^2^/g. Sulphuric acid-activated MMT catalyst was found to exhibit a higher surface area of about 206 m^2^/g which might be ascribed to slight alteration in the layered structure of montmorillonite caused by leaching of Al^3+^ ions or any other impurities by acid treatment. In addition, S-MMT was found to possess larger pore volumes of 0.93 cc/g as compared to MMT which had a pore volume of 0.26 cc/g. The increase in both specific surface area and total pore volume is a crucial element to confirm the process of acid activation^24^. This result corroborates with decrease in (001) reflection found from XRD analysis. However, for platinum loaded catalysts (Pt/S-MMT), the surface area and pore volume were found to be reduced to 198 m^2^/g and 0.79 cc/g when compared to S-MMT catalyst, and a gradual decrease was observed with increase in Pt loading. This attributes to the pore blockage of MMT with Pt or extra framework species.

#### CO-Chemisorption

The platinum dispersion, average particle size and metal surface area has been determined by chemisorption of CO on a series of 0.5–3 wt% Pt/S-MMT catalysts. The results (Supplementary Table S2) demonstrate that as the Pt content in the catalyst increases, the percentage of platinum dispersion was lowered with obvious rise in CO uptake volume. However, the particle size of platinum increased with loading and was found to be in the range of 1.9–4.5 nm. The decrease in dispersion and increase in particle size with Pt loading was probably due to the agglomeration of platinum, promoted by excessive deposition of platinum particles on the surface of MMT, thereby, decreasing the distance between platinum particles. Chary *et al*.^20^ in their study on glycerol hydrogenolysis over Pt-WO_3_/SBA-15 catalysts observed that the addition of tungstate species on to SBA-15 has significantly lowered the Pt dispersion. The results of CO-chemisorption are well correlated with those acquired from XRD and TEM analysis.

The loading of Pt in a series of 0.5–3 wt% Pt/S-MMT catalysts was confirmed by Inductive Coupled Plasma-Atomic Emission Spectrometer (ICP-AES) and the results are presented in Table S2 (Supplementary).

#### Transmission Electron Microscopic Analysis

**Figure 3:**
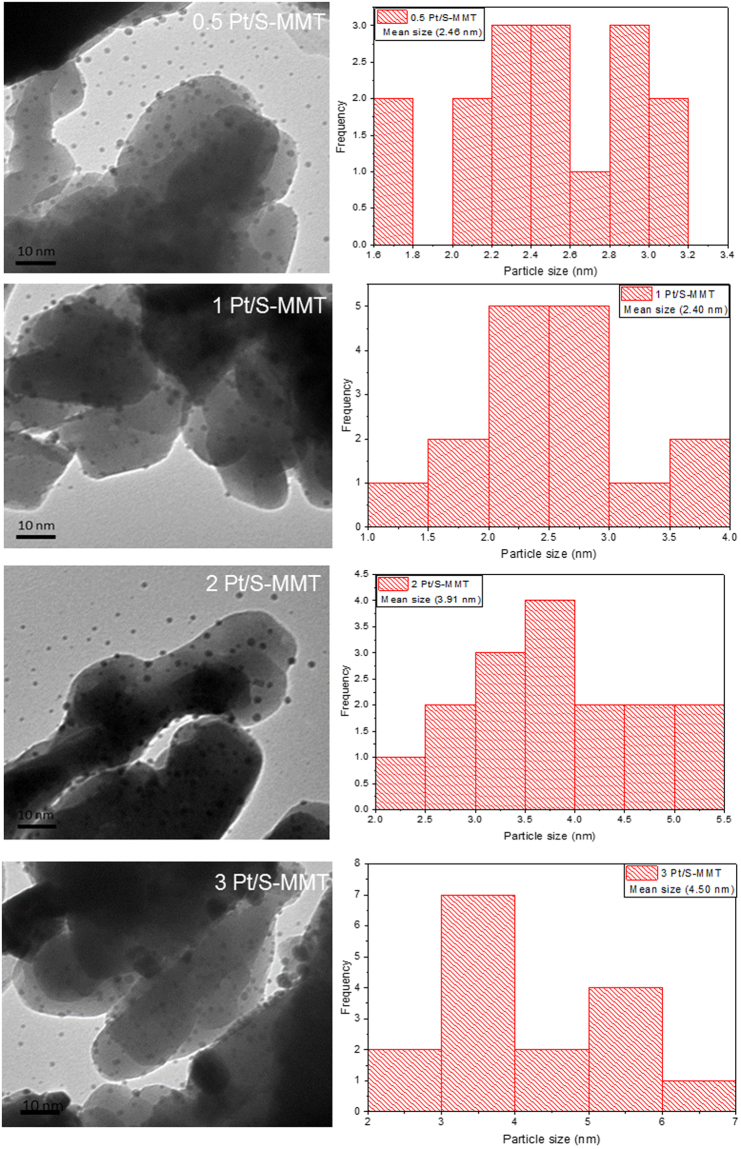
Transmission electron micrographs of different Pt/S-MMT catalysts.

Figure 3 illustrate the TEM micrographs and particle size distribution of the Pt incorporated sulphuric acid-activated montmorillonite catalysts. The images clearly show that the Pt nanoparticles are spherical, fine and well dispersed on the exterior of montmorillonite.The average particle size of platinum in 0.5 wt% and 1 wt% Pt/S-MMT catalysts is found to be around 2.4 nm while platinum size in 2 Pt/S-MMT and 3 Pt/S-MMT catalysts is 3.9 nm and 4.5 nm respectively. At lower loadings, platinum nanoparticles seems to be well dispersed on the support and at higher loadings platinum nanoparticles tend to aggregate forming larger particles. The TEM image of PVP capped platinum nanoparticles is presented in Fig. S4 (Supplementary) and the particle size of platinum nanoparticles is in the range of 2.3–5 nm which is consistent with the sizes observed in supported platinum catalysts.

#### Acidity of catalysts

The acid strength of MMT, S-MMT and 0.5–3 wt% Pt/S-MMT catalysts was determined by ammonia - temperature programmed desorption (NH_3_-TPD) analysis. Based on the temperature regions of ammonia desorption, the acid sites in the catalysts were distinguished as weak (100–200 °C), medium (200–400 °C) and strong (400–700 °C) acid sites. The sum of all the three acid sites measured by the total ammonia desorbed gives the total acidity of the catalysts. The results are compiled in Table 1 and the TPD profile is displayed as Fig. S5 (Supplementary). As shown in the profile, the raw MMT displayed two peaks - one in the low temperature region and the other in high temperature region corresponding to weak and strong acid sites respectively^26^. The desorption of NH_3_ in the low temperature region might be due to the physical adsorption of NH_3_ in the interlayer space of MMT, whereas the NH_3_ desorbed in the high temperature region could be ascribed to the framework Al sites of MMT.

**Table 1:** Acidities of MMT, S-MMT and various Pt/S-MMT catalysts.

Catalyst	NH_3_ uptake (μmol/g)			Total acidities (μmol/g)
	Weak	Moderate	Strong	
MMT	159	—	203	362
S-MMT	224	64	145	433
0.5Pt/S-MMT	143	98	139	380
1Pt/S-MMT	154	43	69	266
2Pt/S-MMT	167	28	50	245
3Pt/S-MMT	122	15	35	172

Interestingly, the NH_3_-TPD profile of S-MMT catalyst showed the presence of an additional peak at moderate temperature region attributing to medium acid sites. This indicates that acid activation of MMT has induced the generation of moderate acid sites in the catalysts. Similarly, three types of acid sites have been identified in Pt/S-MMT catalysts of varying Pt loadings (0.5–3 wt%). It is notable that with increase in the Pt loading, the extent of desorption was shifted more toward lower temperature which means that there was a loss in both moderate and strong acid sites with substantial increase in the weak acid sites. This infers that addition of platinum has facilitated the formation of weak acid sites maximizing at 2 wt% Pt. Furthermore, the total acidity of catalysts was found to decrease with increase in Pt loading. This is because the addition of platinum on sulphuric acid-treated montmorillonite blocks the Brønsted acid sites although marginally increases the Lewis acidity and also due to the possible agglomeration or bulk nature of platinum on support at higher loadings.

**Figure 4:**
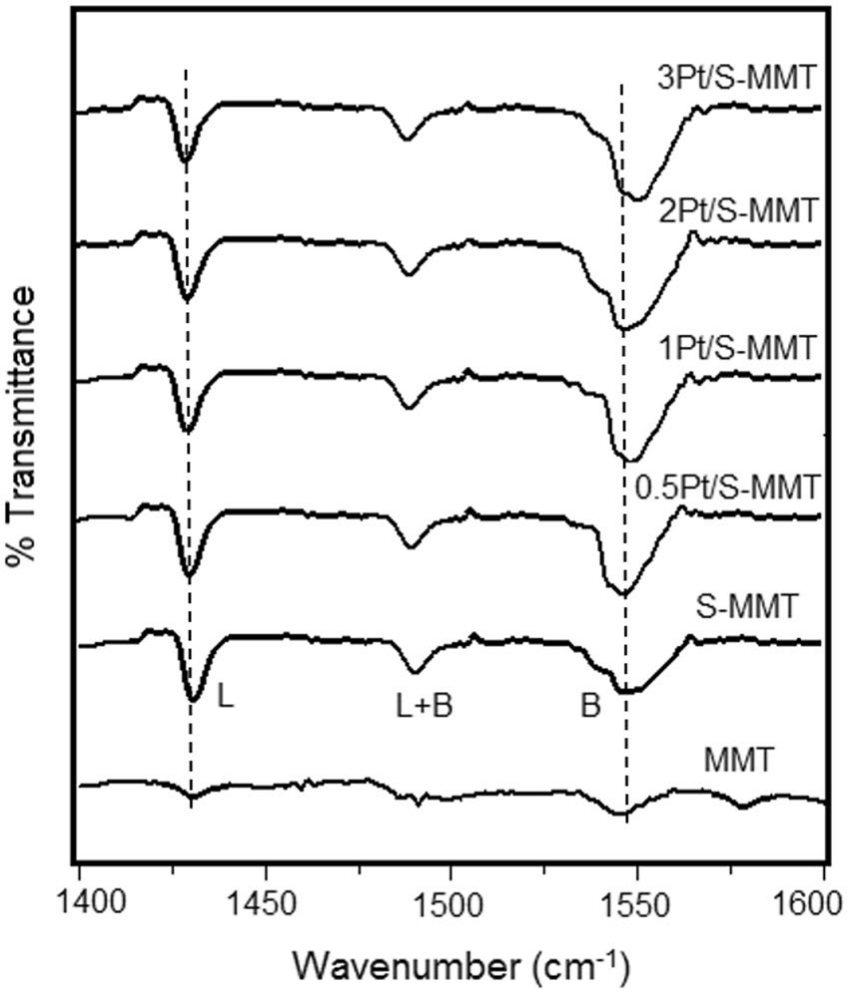
FTIR spectra of pyridine adsorbed MMT, S-MMT and various Pt/S-MMT catalysts.

The presence of Lewis (L) and Brønsted (B) acid sites in MMT, S-MMT and 0.5–3 wt% Pt/S-MMT catalysts was confirmed by FTIR analysis of corresponding pyridine adsorbed species. The vibration frequencies associated to the chemical properties of acid sites are shown in the spectra (Fig. 4). The FTIR spectrum of pyridine adsorbed MMT catalyst did not reveal the occurrence of prominent acid sites which indicates the inherent weak acidity of MMT. Whereas the spectrum of pyridine adsorbed S-MMT catalyst shows well resolved significant bands at 1428 cm^−1^ for pyridine associated to Lewis acid sites; 1480 cm^−1^ due to both Lewis and Brønsted acid sites (total acid sites) and 1540 cm^−1^ due to pyridinium linked to Brønsted acid sites^27^. A similar pattern was observed in the spectra of pyridine adsorbed 0.5–3 wt%) Pt/S-MMT catalysts. The peak intensity corresponding to Brønsted acid sites (at 1540 cm^−1^) in all the catalysts is relatively higher compared to Lewis acid sites indicating that the concentration of Brønsted acid sites is high in the catalysts. The NH3-TPD and Pyr IR results therefore clearly show that the acid activation of MMT has enhanced the surface acidity of the catalysts, and essentially more of Brønsted acid type.

#### SEM-EDX

The topographical features and a semi quantitative elemental analysis of the catalysts has been studied by Scanning Electron Microscopy (SEM) coupled with Energy Dispersive X-ray spectroscopy (EDX). The SEM images and EDX profiles of MMT, S-MMT and 2Pt/S-MMT catalysts are presented in Fig. S6 (Supplementary). The typical SEM image of MMT catalyst displayed larger aggregates (Supplementary Fig. S6a) whereas that of S-MMT showed the development of pores on the surface with slight variation in the crystal morphology^27^. In case of 2Pt/S-MMT catalyst, the aggregates appeared smaller in size compared to MMT and S-MMT catalysts. The elemental analysis data for all the catalysts is listed in Table S3 (Supplementary). For all the catalysts, SiO2 and Al2O3 are the major components, consistent with the main structure of montmorillonite. The element sulpur (S) was identified in the chemical spot analysis of sulphuric acid-activated MMT catalyst. The Pt/S-MMT catalysts revealed the presence of Pt and proves the incorporation of Pt in to the catalysts. As evident from the results, the atomic percentage of Pt in the catalysts corresponded to the Pt loading that was used during the catalyst preparation procedure.

#### ^27^Al NMR spectroscopy

**Figure 5:**
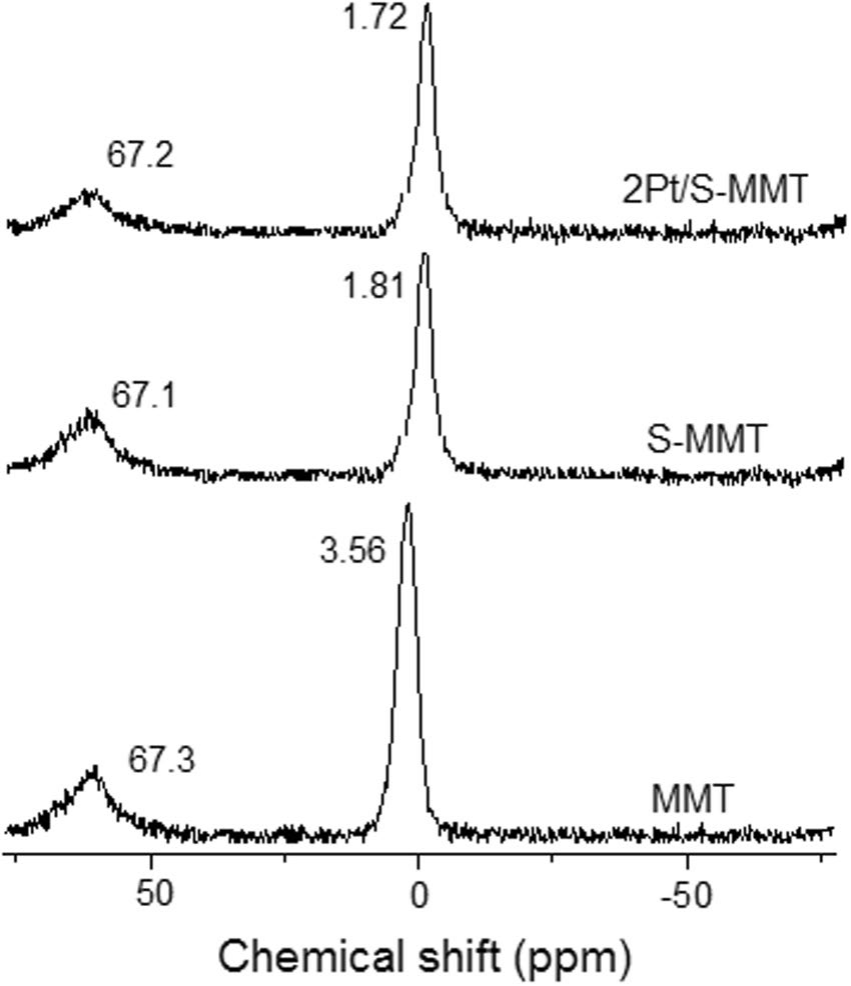
^27^Al NMR spectra of MMT, S-MMT and 2Pt/S-MMT catalysts.

The ^27^Al nuclear magnetic resonance (NMR) spectroscopy has been used to detect the coordination of aluminium within the interlayer structure of montmorillonite. The ^27^Al NMR spectra (Fig. 5) of pure MMT exhibited an intense peak at 3.56 ppm corresponding to octahedral coordinated Al and a small peak at 67.39 ppm due to tetrahedral coordinated Al in the framework of montmorillonite structure. However, acid activation of montmorillonite has resulted in shift of octahedral Al peak to 1.81 ppm with a decreased intensity while the tetrahedral Al peak remained intact^28,29^. This indicates leaching of octahedral Al during acid activation and stable tetrahedral structure of aluminium. The spectra of 2Pt/S-MMT catalyst appeared similar to that of S-MMT catalyst with no considerable change in the chemical shifts.

### Catalytic performance

The hydrogenolysis of glycerol is a multifaceted process in which acid sites and metal sites of the catalyst are indeed the crucial elements for the selective formation of 1,2-PDO and 1,3-PDO. Glycerol conversion to 1,3-PDO was proposed to initiate via Brønsted acid catalyzed dehydration to 3-hydroxypropionaldehyde (3-HPA) followed by metal catalyzed hydrogenation to 1,3-PDO^10,21,30,31^. To establish the validity of this hypothesis, several experiments of glycerol hydrogenolysis were performed using various catalysts including the parent montmorillonite, sulphuric acid-activated montmorillonite and a series of 0.5–3 wt% Platinum on sulphuric acid-activated montmorillonite catalysts to investigate the glycerol conversion and product selectivity. The hydrogenolysis of glycerol was performed in a continuous flow fixed-bed reactor set-up. The reactions were typically carried out at a temperature of 200 °C and a hydrogen pressure of 1 bar using 10 wt% aqueous glycerol and 0.5 g of catalyst. The catalytic activity results for various catalysts are summarized in Table 2. The products from glycerol hydrogenolysis included 1,3-PDO as the major while 1,2-PDO, acrolein, and hydroxyacetone are the secondary products (Supplementary Fig. S7). Other minor products such as acetaldehyde, 1-propanol, 2-propanol and acetone were also detected.

**Table 2:** Catalytic performance of MMT, S-MMT and 0.5–3 wt% Pt/S-MMT catalysts in glycerol hydrogenolysis^a^.

Catalyst	Conversion (%)	Selectivity (%)				
		1,3-PDO	1,2-PDO	Acrolein	HA	Others
MMT	36	—	16.0	35	29	20
S-MMT	70	1.2	2.8	84	8.0	4.0
0.5Pt/S-MMT	79	51	18.9	20	6.0	4.1
1Pt/S-MMT	86	57	16	21	4.3	1.7
2Pt/S-MMT	94	62	12	14	3.1	8.9
3Pt/S-MMT	94	58	13	17	3.0	9.0

^a^Reaction conditions: 10 wt% glycerol aqueous solution; 0.5 g of catalyst; reaction temperature of 200 °C, 1 bar H_2,_ H_2_ flow rate of 70 mL min^−1^; 1,3-PDO: 1,3-propanediol, 1,2-PDO: 1,2-propanediol, HA: Hydroxyacteone, Others include acetaldehyde, 1-propanol, acetone, 2-propanol.

When the parent MMT was tested in glycerol hydrogenolysis, the glycerol conversion was fairly low with acrolein being the major product and substantial amounts of degradation products. Sulphuric acid-activated MMT catalyst resulted in increase in glycerol conversion of 70% with a high selectivity to acrolein at around 84% and less selectivity to propanediols (~3%). This infers that the acid treatment of MMT resulted in generation of high strength Brønsted acidic sites in the catalyst, evident from Pyr IR, and has enabled the double dehydration of glycerol to produce acrolein. A similar finding has been reported by Zhou *et al*^24^. The different platinum catalysts were then tested for their activity for glycerol transformation to 1,3-PDO. It is worth noting that the glycerol conversion and selectivity to 1,3-PDO was greatly improved over sulphuric acid-activated montmorillonite supported platinum catalysts compared to the parent MMT and S-MMT.

#### Influence of Pt loading

To gain an insight into the 1,3-PDO selectivity, the effect of platinum loading (0.5–3 wt%) was studied under the standard reaction conditions. As the Pt loading is increased from 0.5–2 wt%, there was a gradual increase in the glycerol conversion from 79% to 94% and selectivity to 1,3-PDO from 51% to 62% (Table 2). Over 3 wt% Pt/S-MMT catalyst, the selectivity to 1,3-PDO marginally reduced while the glycerol conversion remained the same. The maximum glycerol conversion and selectivity to 1,3-PDO was achieved over 2Pt/S-MMT catalyst, meaning that 2 wt% Pt was adequate to hydrogenate 3-HPA formed over S-MMT, to 1,3-PDO. Therefore, among all the other catalysts screened, 2 wt% Pt/S-MMT was found to be the optimal and most active catalyst for glycerol transformation to 1,3-PDO, under the standard conditions, and hence chosen for further reaction optimization experiments. Similar findings on the predominant effect of 2 wt% Pt in glycerol hydrogenolysis have been demonstrated earlier^32,33^.

#### Reaction parametric study over 2Pt/S-MMT catalyst

**Figure 6:**
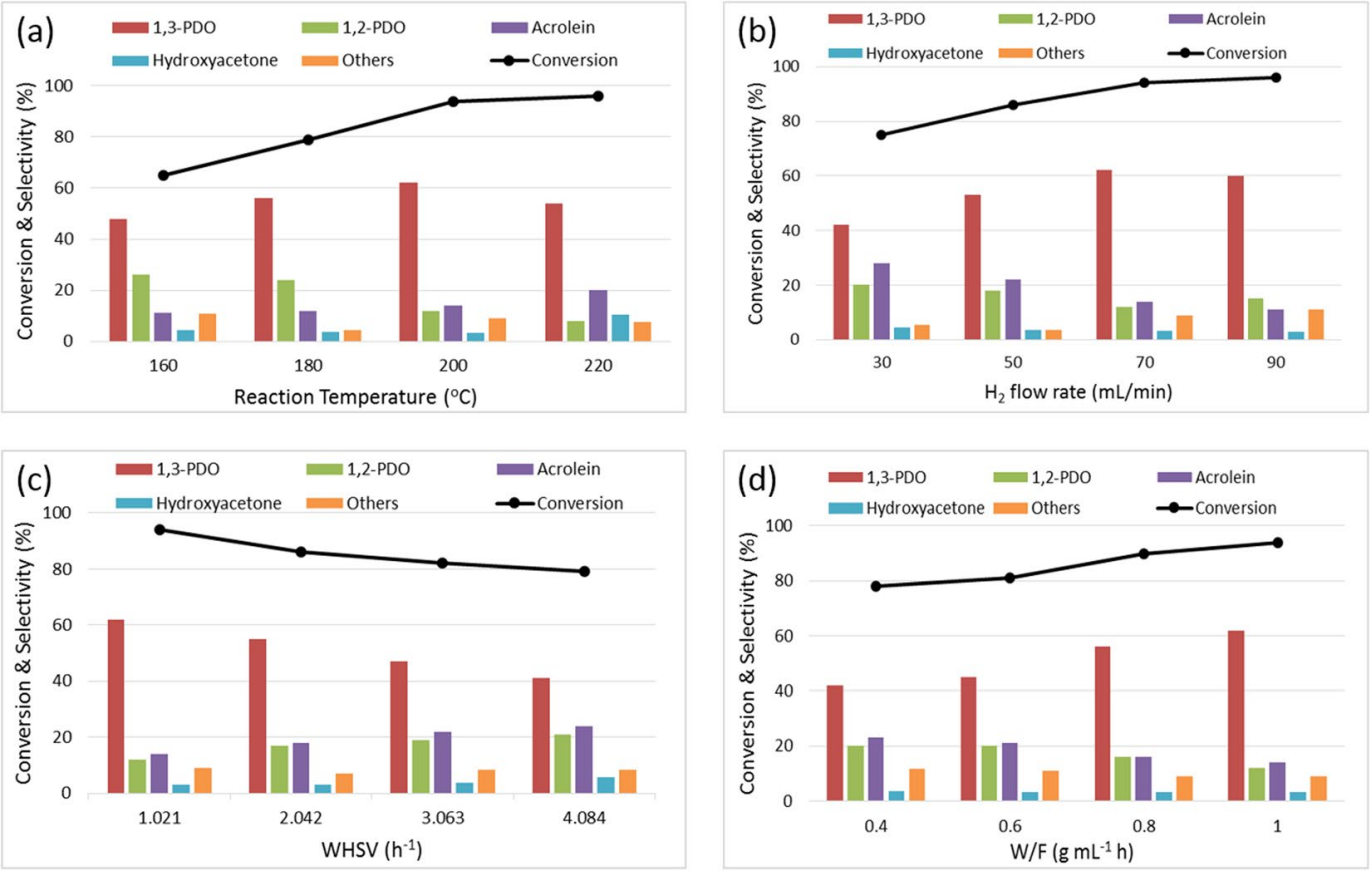
Effect of reaction temperature (**a**), hydrogen flow rate (**b**), weight hourly space velocity (**c**) and contact time (**d**) on glycerol conversion and product selectivity over 2Pt/S-MMT catalyst. Reaction conditions: 10 wt% glycerol aqueous solution; 0.5 g of catalyst;, 1 bar H_2_; 1,3-PDO: 1,3-propanediol, 1,2-PDO: 1,2-propanediol, HA: Hydroxyacteone, Others include acetaldehyde, 1-propanol, acetone, 2-propanol.

The effect of reaction temperature: Figure 6a represents the results of hydrogenolysis of glycerol over 2Pt/S-MMT catalyst conducted at different reaction temperatures ranging from 160–220 °C and atmospheric pressure. As the reaction temperature elevated from 160 °C to 220 °C, there was a steady increase in the glycerol conversion from 65% to 96%. The selectivity to 1,3-PDO gradually increased until a reaction temperature of 200 °C beyond which it slightly lowered. On contrary, a steady decrease in 1,2-PDO selectivity has been observed when the reaction temperature accelerated. This is ascribed to the fact that higher reaction temperatures would stimulate additional carbon-oxygen and carbon-carbon bond scission in glycerol leading to other byproducts such as propanols, hydroxyacetone, acrolein and acetaldehyde with decreased 1,3-PDO selectivity. This is in accordance with the previous reports^31,34^. 2Pt/S-MMT catalyst presented almost 94% conversion of glycerol and a maximum of 62% selectivity to 1,3-PDO at 200 °C, considering it to be the appropriate and optimum reaction temperature to promote glycerol hydrogenolysis.

The effect of of hydrogen flow rate: Several experiments were carried out to check the effect of hydrogen flow rate on hydrogenolysis of glycerol at 200 °C and different H_2_ flow rates of 30, 50, 70 & 90 mL min^−1^ over 2Pt/S-MMT catalyst. The results are summarized in Fig. 6b. As the hydrogen facilitates glycerol hydrogenolysis reaction, it is obvious that, with rise in H_2_ flow rate, there happened a steep rise in the glycerol conversion. While 1,3-PDO selectivity has gradually increased with an increase in H_2_ flow rate from 30 to 70 mL min^−1^, it must be noted that, increasing H_2_ flow rates suppressed the formation of 1,2-PDO and acrolein favoring 1,3-PDO from glycerol. However, further rise to 90 mL min^−1^ H_2_ flow rate, the selectivity to 1,3-PDO dropped a bit though the glycerol conversion increased. A possible explanation is that high H_2_ flow rate probably promoted the formation of corresponding excessive glycerol hydrogenolysis products such as ethanol, 1-propanol, 2-propanol and acetaldehyde. Thus, for both glycerol conversion and 1,3-PDO selectivity, the optimal H_2_ flow rate was 70 mL min^−1^. This trend of results is in perfect agreement with the past reports^31,35^.

The effect of weight hourly space velocity (WHSV): The flow rate of glycerol is expected to have a significant effect on the glycerol hydrogenolysis and to examine this, reactions over the most active catalyst were carried out at 200 °C by changing the WHSV of 10 wt% glycerol from 1.02–4.08 h^−1^. Figure 6c showed that with rising WHSV, the glycerol conversion decreased significantly from 94% to 79%, together with a steady decline of 1,3-PDO selectivity from 62% to 41%. This suggests that as the glycerol feed flow increases, the number of active sites to react with glycerol decreases and most likely not adequate to convert excess glycerol^32^. The other product distribution showed that the selectivity towards acrolein and 1,2-PDO showed uniform increase with the continuously increasing WHSV. Thus, the results showed that the lower WHSV (1.02 h^−1^) was indeed efficient to promote the selective glycerol hydrogenolysis to 1,3-PDO with a maximum selectivity of 62% at 94% glycerol conversion.

The effect of contact time (W/F): The ratio of catalyst weight to the feed flow rate is important to optimize the reaction process and to achieve maximum product selectivity and reactant conversion. To understand the effect of contact time (W/F) on the activity and selectivity, experiments were run by varying the weight of the catalyst at constant glycerol feed flow rate. As shown in the Fig. 6d, the selectivity to 1,3-PDO and glycerol conversion and enhanced steadily with increase in the contact time. This is because, higher the contact time, greater is the interaction between the catalyst active sites and the glycerol that would favor the selective production of 1,3-PDO and simultaneously increase glycerol conversion. However, the opposite trend was observed in case of 1,2-PDO and acrolein selectivity. The results conclude that a higher contact time of 1.0 g mL^−1^ h was found to be ideal for attaining the maximum glycerol conversion and 1,3-PDO selectivity. The observation is consistent with the literature^20^.

#### Space Time Yield (STY)

The quantity of 1,3-PDO produced per 0.5 g of 2Pt/S-MMT catalyst per one hour of reaction time refers to the space time yield (STY) of 1,3-PDO. The STY of 1,3-PDO has been studied by varying the glycerol concentration (5–20 wt%) of the experiments performed at a reaction temperature of 200 °C, 70 mL/min H_2_ flow rate and atmospheric pressure. The following equation was employed to calculate the space time yield (STY) of 1,3-PDO:

$${STY}={{M}}_{1{,}3 \mbox{-} {PDO}}/{{M}}_{{cat}}\times {t}$$

where M_1,3-PDO_ is the mass of 1,3-PDO produced (g); M_cat_ is the mass of catalyst (g); and t is the reaction time (h). The space time yield towards 1,3-PDO is plotted as a function of glycerol concentration and the results are presented in Fig. S8 (Supplementary). As can be seen from the plot, as the glycerol concentration rises from 5 to 20 wt%, the space time yield towards 1,3-PDO and glycerol conversion increased up until 10 wt% after which a fall has been observed. With increase in the glycerol content (beyond 10 wt%), the viscosity of the feed increases which might reduce the mass transfer performance of the glycerol and 1,3-PDO on the catalyst surface^36^, thereby lowering 1,3-PDO selectivity and the glycerol conversion. The optimal glycerol concentration for the reaction was found to be 10 wt% when the glycerol conversion and the 1,3-PDO selectivity were considered together.

#### Glycerol hydrogenolysis reaction mechanism over Pt/S-MMT catalyst

**Figure 7:**
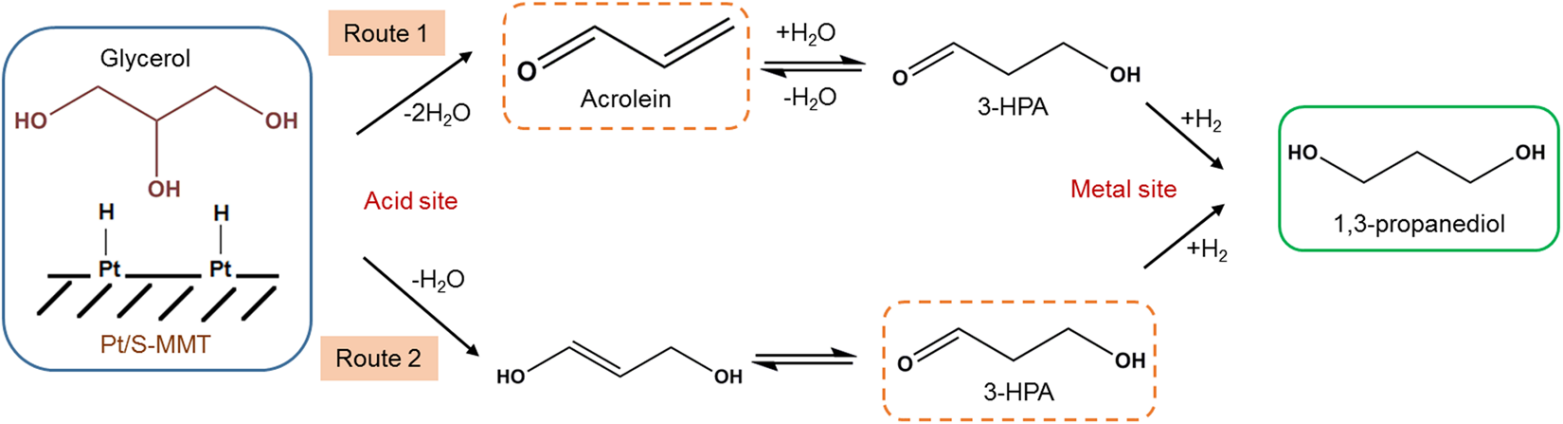
The possible routes of 1,3-PDO formation in vapour phase glycerol hydrogenolysis over Pt/S-MMT catalyst in a fixed bed reactor under atmospheric pressure.

The structure-activity correlation indicated that the glycerol hydrogenolysis to 1,3-PDO reaction over a bifunctional Pt/S-MMT catalyst proceeds through a dehydration-hydrogenation mechanism in two different routes. As presented in Fig. 7, Route 1 is a three-step process in which glycerol undergoes double dehydration over Brønsted acidic sites of S-MMT to produce acrolein and the rehydration of acrolein gives 3-hydroxypropionaldehyde (3-HPA) which then hydrogenates over platinum sites to produce 1,3-PDO. In Route 2, 1,3-PDO is formed via two-step dehydration of glycerol to 3-HPA and its subsequent hydrogenation to 1,3-PDO. A similar mechanism has been proposed in recent work published by Mahesh *et al*^37^.

#### Catalyst stability and reusability

The stability of 2Pt/S-MMT catalyst with time-on-stream over was studied for a period of 1–8 h reaction under the optimized reaction conditions as follows: reaction temperature: 200 °C; glycerol concentration = 10 wt.%, WHSV = 1.02 h^−1^, and H_2_ flow rate = 70 mL min^−1^. It is observed that there is a gradual rise in glycerol conversion from 86% to 94% during 1–3 h and then remained unchanged until 5 h; after which it declined slowly to 80% (Supplementary Fig. S9a). A similar trend was observed in case of 1,3-PDO selectivity, during 8 h period with a maximum selectivity of 62% being achieved at 3 h. The decline in the activity during the latter hours could be probably due to the deactivation of the catalyst which was probably the result of glycerol polymerization or further hydrogenolysis of 1,3-PDO on the catalyst. To confirm this, the spent catalyst was re-activated at 500 °C for 3 h in an air flow (100 mL min^−1^), to remove the carbon species deposited on the catalysts. The regenerated/reactivated catalyst (labelled as 2Pt/S-MMT R) was then tested in a second time-on-stream studies (Supplementary Fig. S9b). The results showed a better recovery of the catalytic performance, with a similar deactivation trend as that of the fresh catalyst. However, there was a little drop in the glycerol conversion (90%) and 1,3-PDO selectivity (60%) over the regenerated catalyst. The drop in the activity could be due to the change in spent catalyst morphology^38^ including decrease in the total acidity and BET surface area, verified by NH_3_-TPD and BET SA analyses; pore blockage by coke deposition, as shown from CHNS analysis (Supplementary Table S4). The acidity of spent (deactivated) catalyst was found to be considerably lower than that of fresh catalyst which is due to the blockage of acid sites with carbonaceous species. However, it is notable that the reactivated catalyst showed a marginal decrease in the acidity to that of fresh catalyst and has resulted in the better catalytic performance. Therefore, NH_3_-TPD results suggests that the acidity of a catalyst plays a crucial role in determining the catalytic activity for hydrogenolysis of glycerol^21^. The thermogravimetric analysis (TGA) of fresh and spent 2Pt/S-MMT catalysts was performed at 10 °C/min over a temperature range of 50 °C to 750 °C under a constant flow of nitrogen (20 mL/min) to further understand the mode of catalyst deactivation. The degradation pattern of fresh and spent 2Pt/S-MMT catalysts (Supplemenatry Figure S10) showed two well-defined mass loss regions; the first one between 50 °C and 200 °C is attributed to the loss of physisorbed water from the interlayer spacing of montmorillonite and the second mass loss above 400 °C. is due to the dehydration of coordinated water molecules and dehydroxylation of silicate structure. However, a total of about 23 wt% mass loss was observed for the spent catalyst over the range of 50 °C to 750 °C, compared with only <5 wt% mass loss for the fresh catalyst. This result is a clear evidence of the coke formation and deposition of undesired materials due to glycerol polymerization on the spent catalyst, which contributed to the catalyst deactivation by blocking the catalyst active sites.

## Discussion

This research work demonstrated a novel approach of catalyst design comprising of 0.5–3 wt% Pt nanoparticles immobilized on sulphuric acid-activated montmorillonite, investigated first time for the selective production of 1,3-PDO by hydrogenolysis of biomass derived glycerol. The activation of montmorillonite by sulphuric acid did not obviously alter the inter layer structure of montmorillonite, as evidenced from XRD, FTIR and ^27^Al NMR but enhanced the acid catalytic performance by generation of new acid sites preferentially of Brønsted acid type, revealed from NH_3_-TPD and Pyr IR analyses. Among the various tested catalysts, 2Pt/S-MMT catalyst exhibited superior performance with 62% selectivity to 1,3-PDO at 94% glycerol conversion under reasonably mild reaction conditions. The results from efficient characterization and the catalytic experiments clarify that Brønsted acid sites generated by acid activation of the catalyst promoted selective abstraction of secondary –OH group of glycerol favoring the formation of 1,3-PDO and the highly dispersed nanosize Pt particles, confirmed from CO-chemissorption and TEM studies, enabled the hydrogenation of 3-hydroxypropanal. The catalytic performance varied based on the reaction conditions and upon optimization, the favorable reaction conditions to accomplish the best result over the most active 2Pt/S-MMT catalyst, were found to be a reaction temperature of 200 °C, 0.1 MPa H_2_, 70 mL min^−1^ H_2_ flow rate, a WHSV of 1.02 h^−1^ and a glycerol concentration of 10 wt%. The catalyst offered good stability and was successfully reused in the consecutive cycle without a larger decline in the activity.

## Methods

### Catalyst Preparation

#### Preparation of sulphuric acid-activated montmorillonite catalyst

The montmorillonite clay (henceforth designated as MMT) obtained from Bentonite deposit, China, was used as received without further purification (BET-surface area ~183 m^2 ^gm^−1^). Concentrated H_2_SO_4_ (98.0%, Sigma Aldrich Co., Ltd) was used to activate montmorillonite clay. The dried montmorillonite powder was activated in an aqueous solution of 10 wt% sulfuric acid at 80 °C with constant stirring for 4 h. After reaction, the acid-activated solid was separated by filtration, then washed three times with distilled water, and air-dried at 100 °C for 48 h^39^. The solid was then ground to fine powder (30 µm) for further use and labeled as S-MMT.

#### Preparation of platinum nanoparticles

Pt nanoparticles (NPs) with an average size of 2.5–5 nm were synthesized following the procedure reported elsewhere^40^. In a typical synthesis, tetraammine platinum (II) nitrate [Pt(NH_3_)_4_(NO_3_)_2_] was used as a Pt precursor and a polymer poly(vinylpyrrolidone) (PVP) was employed as a capping agent. Various loadings (0.5, 1, 2 & 3 wt %) of Pt NPs were prepared by dissolving required amount of Pt in ethylene glycol and PVP. The solution could boil at solvent temperatures. The as-synthesized PVP-capped Pt NPs were then washed and dispersed in ethanol to give a colloidal solution of Pt NPs.

#### Preparation of sulphuric acid-activated montmorillonite supported platinum catalysts

For immobilizing Pt NPs on S-MMT, the colloidal solution of 0.5–3 wt % Pt NPs was added to the S-MMT under stirring. The colloidal suspension was then sonicated for 5 h at room temperature using a UC25-12TPA Ultrasonic cleaner. The resultant solution is centrifuged to obtain brown precipitates, washed with ethanol. Later, the precipitates were dried in an oven at 80 °C overnight, followed by calcination in a muffle furnace at 500 °C for 3 h. All the prepared catalytic materials are hereafter labelled as 0.5Pt/S-MMT, 1Pt/S-MMT, 2Pt/S-MMT, and 3Pt/S-MMT where the number represents the weight percentage (wt%) of Pt.

### Catalyst characterization

The crystallinity and identification of catalyst phases was studied by powder X-ray diffraction (XRD) analysis performed on Rigaku’s Miniflex X-ray diffractometer by means of Ni filtered Cu Kβ radiation (λ = 1.392 Å). The measurements were recorded in the 2θ range of 2 to 80°, scanning at 2° min^−1^. A beam current of 15 mA and a beam voltage of 40 kV were used for the measurements.

The FTIR spectra of catalysts were recorded in transmission mode on a UATR Perkin Elmer Spectrum Two instrument. Spectra were acquired in the spectral range of 4000–400 cm^−1^ at a resolution of 4 cm^−1^ and accumulation of 32 scans. For the analysis of Brønsted and Lewis acid sites in the catalysts, infrared spectra of pyridine adsorbed catalysts (Pyr IR) was acquired in the range of 1600–1400 cm^−1^. For the pyridine adsorption, the catalysts undergo an hour pretreatment under N_2_ at 300 °C for moisture removal followed by adsorption of pyridine at 120 °C for further hour until saturation. The catalysts were then cooled down to 30 °C and pelletized with KBr to record the spectra.

The amount of Pt in all Pt/S-MMT catalysts was quantitatively analyzed by Inductively coupled plasma atomic emission spectrometry (Agilent Technologies-4200MP-AES). The sample preparation includes acid digestion of catalyst (~10 mg in 2 mL aquaregia) at 60 °C and dilution to desired concentration.

Nitrogen adsorption-desorption isotherms were acquired on Quantachrome Autosorb 1 instrument by degassing the samples at 250 °C for 6 h under vacuum and carrying out the analysis at −196 °C under liquid nitrogen. The specific surface areas of catalysts were calculated using the multi-point Brunauer–Emmet–Teller (BET) method. The average pore diameter and pore volumes were measured by adsorption curve analysis using the Barrett–Joyner–Halenda model (BJH) method.

The strength of acid sites in raw MMT, S-MMT and Pt/S-MMT catalysts were determined by NH_3_-TPD measurement using Autochem 2910 (Quantachrome) fitted with a thermal conductivity detector. The samples were prepared by outgassing at 200 °C for 1 h under He flow (50 mL min^−1^). NH_3_ gas was passed through the catalyst until complete saturation for about 30 min and then physisorbed NH_3_ was removed by passing He (50 mL min^−1^) for another 30 min. NH_3_-TPD analysis for continuous monitoring of desorbed NH_3_ was carried out by heating the samples in the temperature range from 100–700 °C at 10 °C min^−1^ under He flow (50 mL min^−1^). The volume of desorbed NH_3_ was measured using GRAMS/32 software.

The morphology and elemental investigation of the catalysts were performed using a Phenom XL Scanning Electron Microscopy coupled with an Energy Dispersive X-Ray Analyzer (SEM-EDX). Each catalyst sample was deposited on a carbon tape adhered to an aluminum stub prior to analysis by SEM. The micrographs of the catalysts were captured at 10 kV beam voltage and 5,000× magnification using a backscatter electron detector (BSD). The elemental analysis was performed at high resolution (15 kV of beam voltage) and high vacuum pressure (1 Pa) with a secondary electron detector (SED), where the point and mapping analysis for element identification was performed using a Phenom Pro Suite software.

The percentage of platinum dispersion was measured by CO-chemisorption experiments typically carried out in an AutoChem 2910 instrument (Micromeritics) associated with a thermal conductivity detector. Average particle size was also verified during the CO-chemisorption measurements. The experiment begins with reduction of 100 mg of the sample at 300 °C under H_2_ flow (50 mL min^−1^) for 3 h, followed by He treatment at 300 °C for 1 h after which the sample is brought to 30 °C. A number of pulses of 9.96% CO balanced helium were injected in regular intervals over the reduced catalyst to measure the volume of CO uptake by the catalyst.

The skeletal aluminium framework structure of montmorillonite catalysts was examined using ^27^Al MAS NMR technique performed on DD2 Oxford Magnet AS-500MHz spectrometer (Agilent Technologies) using aluminium oxide (Aldrich) as a reference material. The chemical shifts (δ) are mentioned in ppm.

The microstructure of Pt catalysts was determined by Transmission electron microscopy (TEM) performed on JEOL 2010 electron microscope operating at 200 KV. A thin catalyst suspension is made by ultra-sonicating tiny amount of powder sample in ethanol and a drop of it is dispersed on copper grids. Samples were positioned in the microscope column, at evacuation of less than 1 × 10^−6^ Torr.

The thermogravimetric analysis (TGA) of the fresh and spent catalysts was performed on a Perkin Elmer TGA 7 unit. The measurements were performed over a temperature range of 50 °C to 750 °C at 10 °C/min under a constant flow of nitrogen (20 mL/min).

### Reaction experiment

The evaluation of catalysts in the hydrogenolysis of glycerol was performed in an upright continuous-fed fixed bed quartz reactor with dimensions of 9 mm internal diameter and 40 cm length. All reactions were carried out in vapour phase under atmospheric pressure. For each experiment, 0.5 g of catalyst mixed with quartz beads was loaded into the middle portion of the reactor and the temperature at the catalyst bed was monitored by a thermocouple. The catalyst bed was heated to 350 °C at an incremental rate of 10 °C min^−1^ in a flow of pure H_2_ (50 mL min^−1^) to perform catalyst reduction for about 2 h. The temperature of the reactor was then lowered to the required reaction temperature and an aqueous solution with a ratio of glycerol to water at 10:90 (w/w) was pumped into the reactor at a flow rate of 0.5 mL h^−1^ using a B-Braun syringe pump together with a stream of H_2_ at a flow rate of 70 mL min^−1^. The unreacted glycerol and the reaction products were condensed and collected every hour in a cold trap (0 °C). The collected samples were analyzed using a gas chromatography (Shimadzu GC 2014) equipped with an FID and DB-wax capillary column (Agilent, 0.32 mm × 32 m). The conversion of glycerol and selectivity to products were calculated using the following equations:

1 $${\rm{Conversion}}\,( \% )=({\rm{moles}}\,{\rm{of}}\,{\rm{glycerol}}\,{\rm{reacted}})/({\rm{moles}}\,{\rm{of}}\,glycerol\,{\rm{fed}})\times {\rm{100}}$$

2 $${\rm{Selectivity}}\,( \% )=({\rm{moles}}\,{\rm{of}}\,{\rm{one}}\,{\rm{product}})/({\rm{moles}}\,{\rm{of}}\,{\rm{all}}\,{\rm{products}})\times {\rm{100}}$$

### Data Availability

The datasets generated during and/or analysed during the current study are available from the corresponding author on reasonable request.

## Electronic supplementary material

Supplementary Information
