# Supplementary material for: Turning Biodiesel Waste Glycerol into 1,3-Propanediol: Catalytic Performance of Sulphuric acid-Activated Montmorillonite Supported Platinum Catalysts in Glycerol Hydrogenolysis
Source: Sci Rep. 2018 May 10;8:7484. doi: 10.1038/s41598-018-25787-w (PMC5945670; doi:10.1038/s41598-018-25787-w)
Supplement: Supplementary file 1 — Supplementary Information [file 41598_2018_25787_MOESM1_ESM.pdf]

Supplementary Information

Turning Biodiesel Waste Glycerol into 1,3-Propanediol: Catalytic Performance of Sulphuric acid-Activated Montmorillonite Supported Platinum Catalysts in Glycerol Hydrogenolysis

Shanthi Priya Samudrala[1*], Shalini Kandasamy[1], Sankar Bhattacharya[1]

[1] Department of Chemical Engineering, Monash University, Melbourne 3800 Australia.

[*]Corresponding author email: priya.shanthipriya@monash.edu

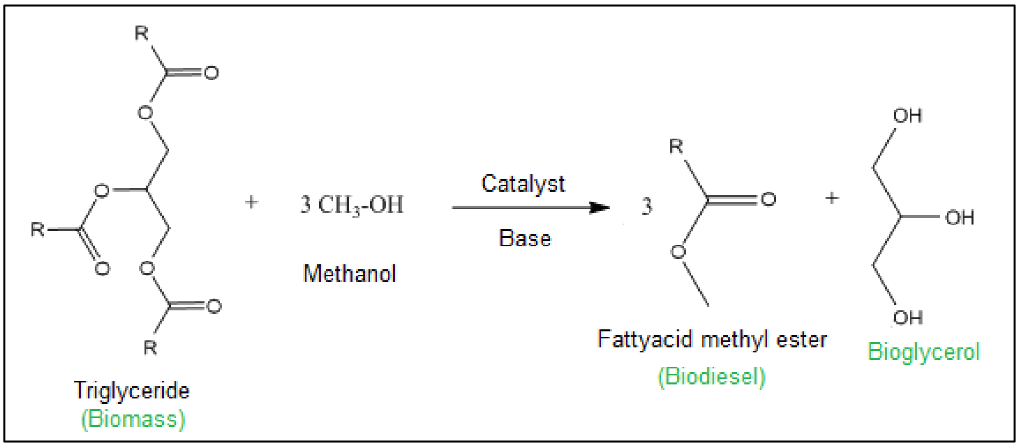

Figure S1. Glycerol as a byproduct in biodiesel production process.

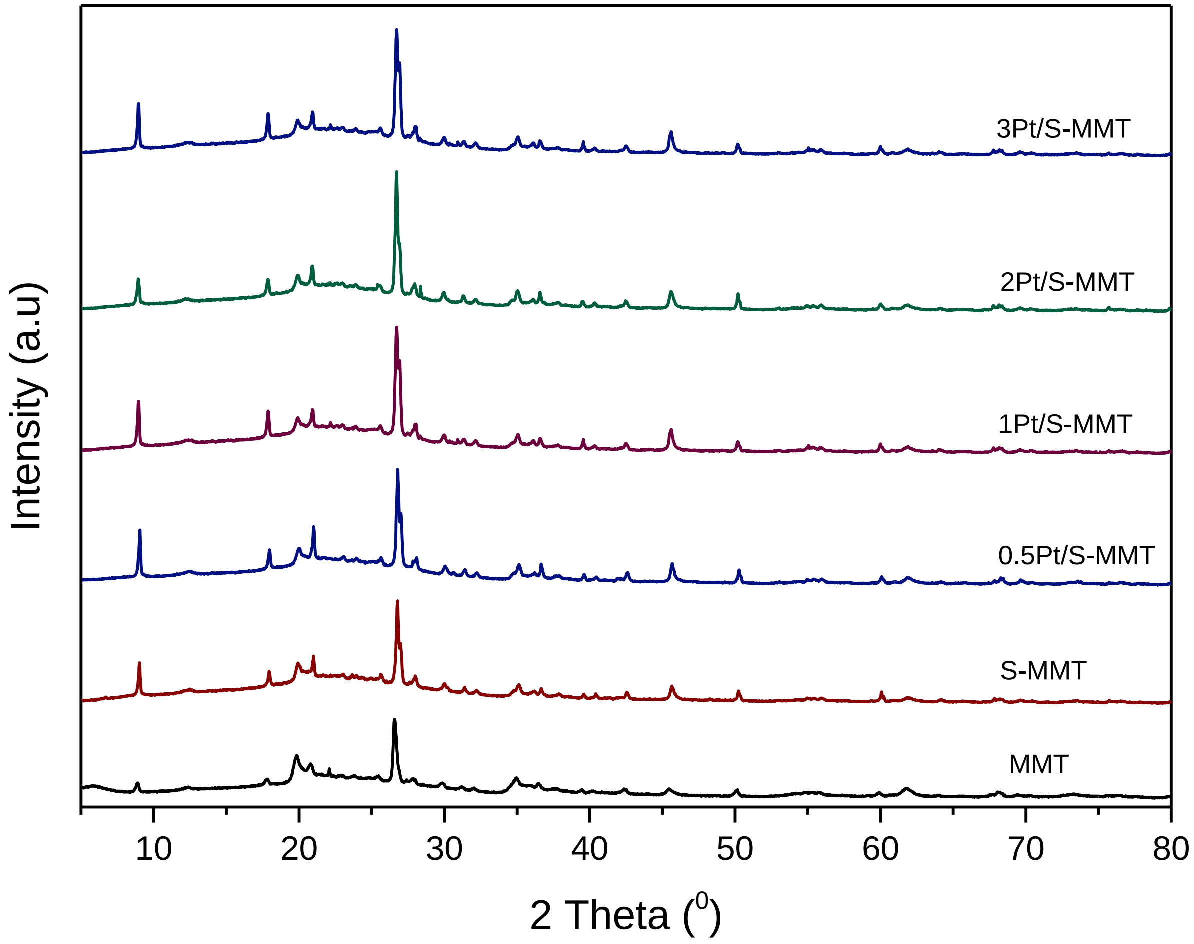

Figure S2. XRD patterns of MMT, S-MMT and different Pt/S-MMT catalysts.

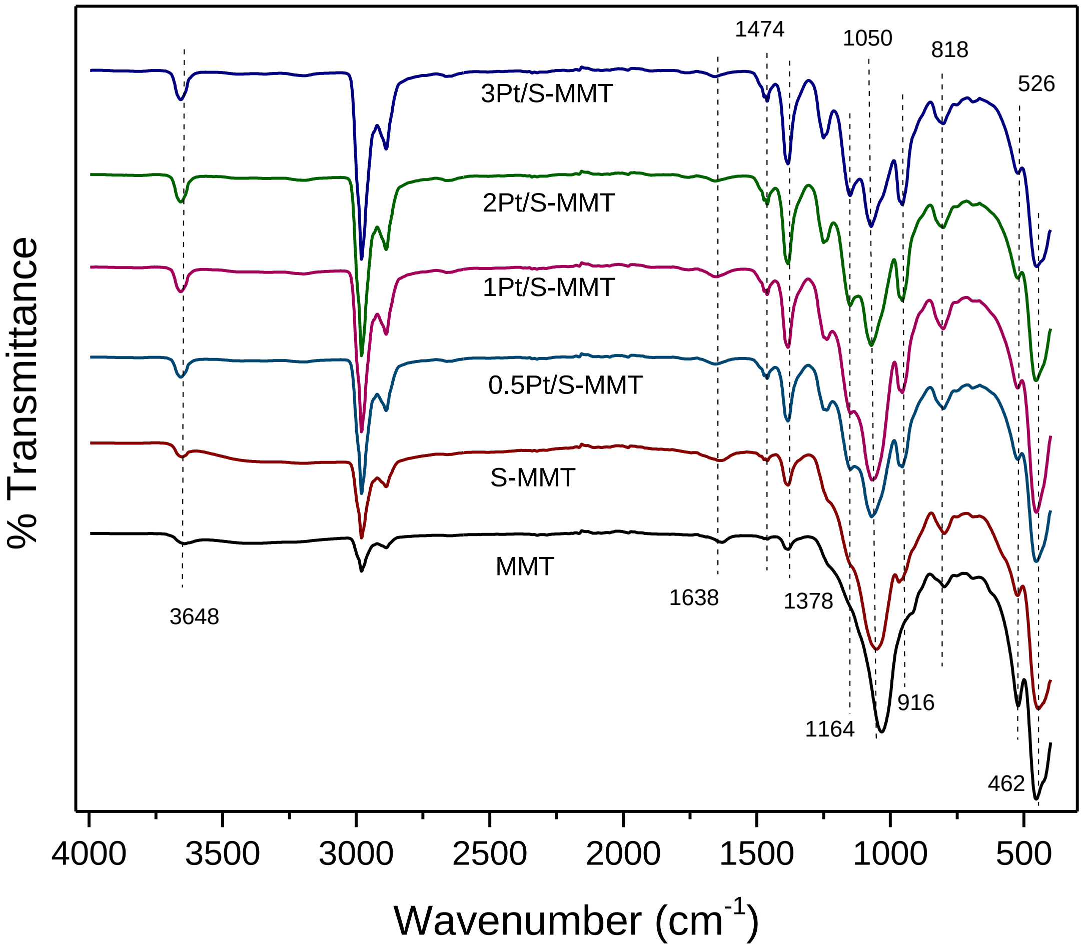

Figure S3. FTIR spectra of MMT, S-MMT and different Pt/S-MMT catalysts.

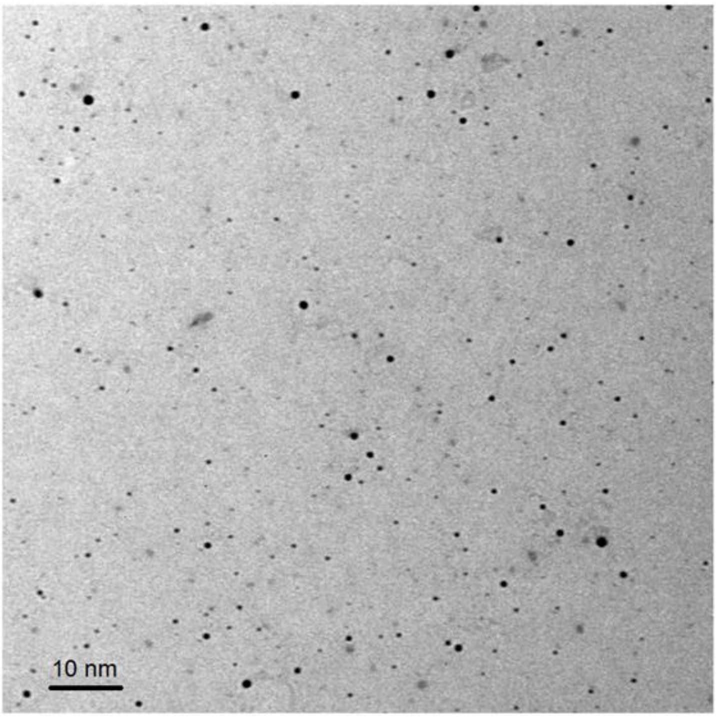

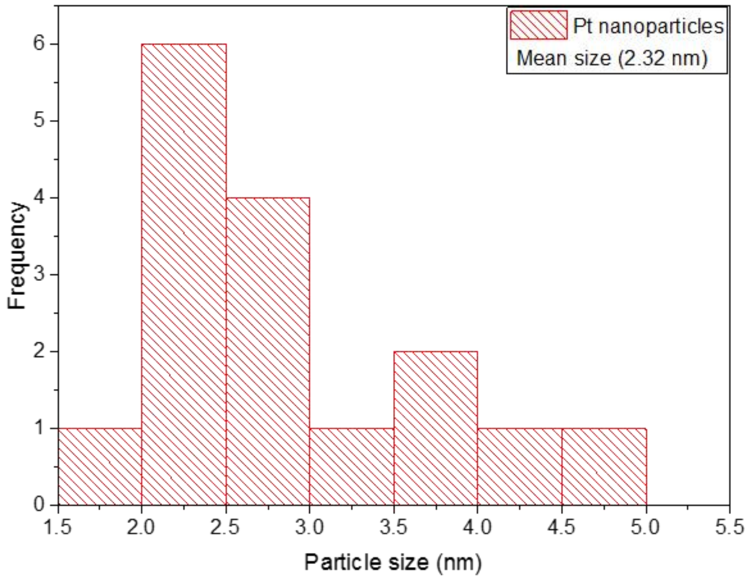

Figure S4. TEM image and particle size distribution of PVP capped Pt nanoparticles

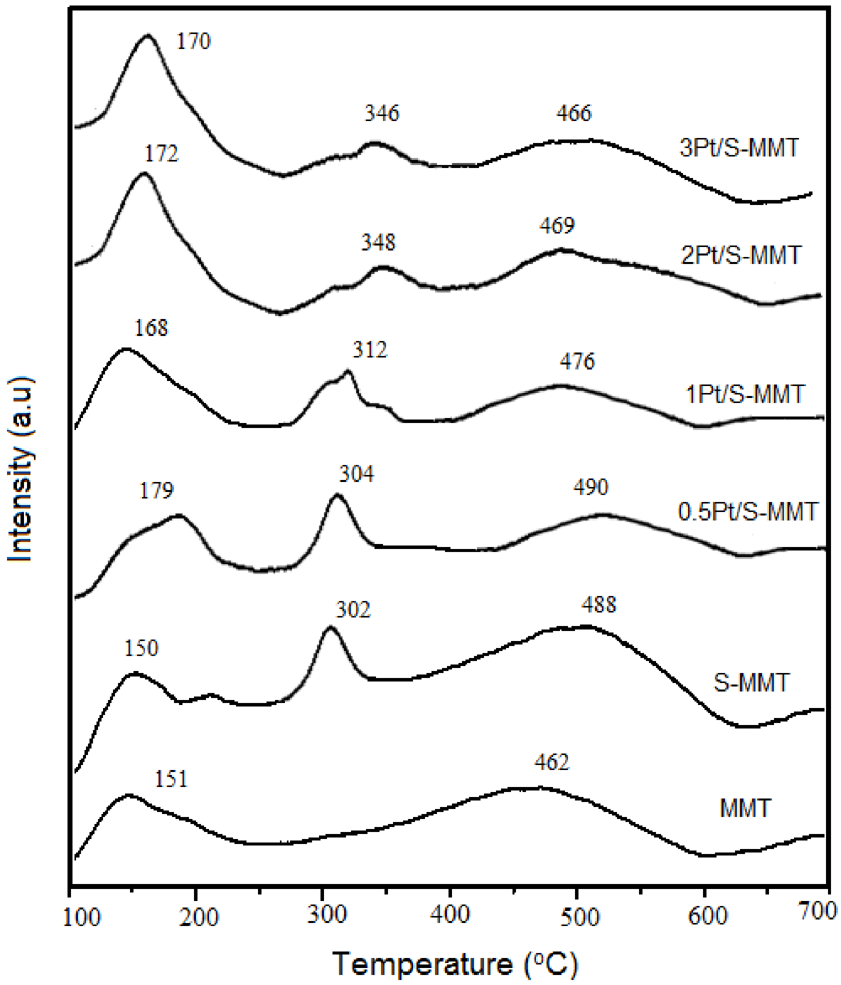

Figure S5. NH_3-TPD profile of pure MMT, S-MMT and various Pt/S-MMT catalysts.

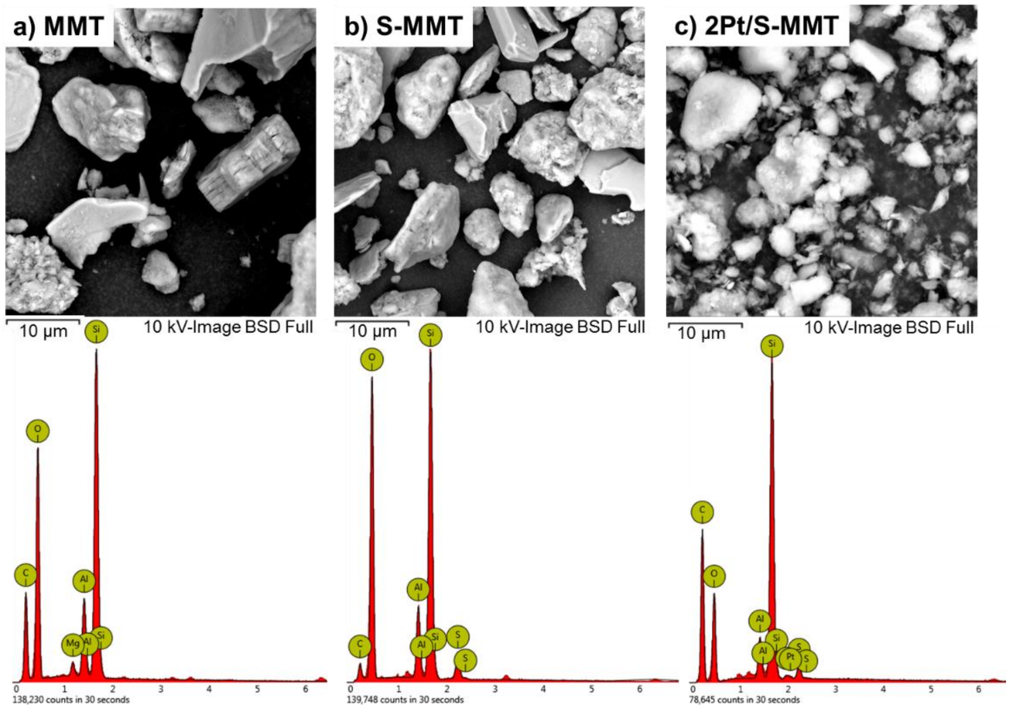

Figure S6. SEM images and EDX spectra of MMT, S-MMT and 2Pt/S-MMT catalysts.

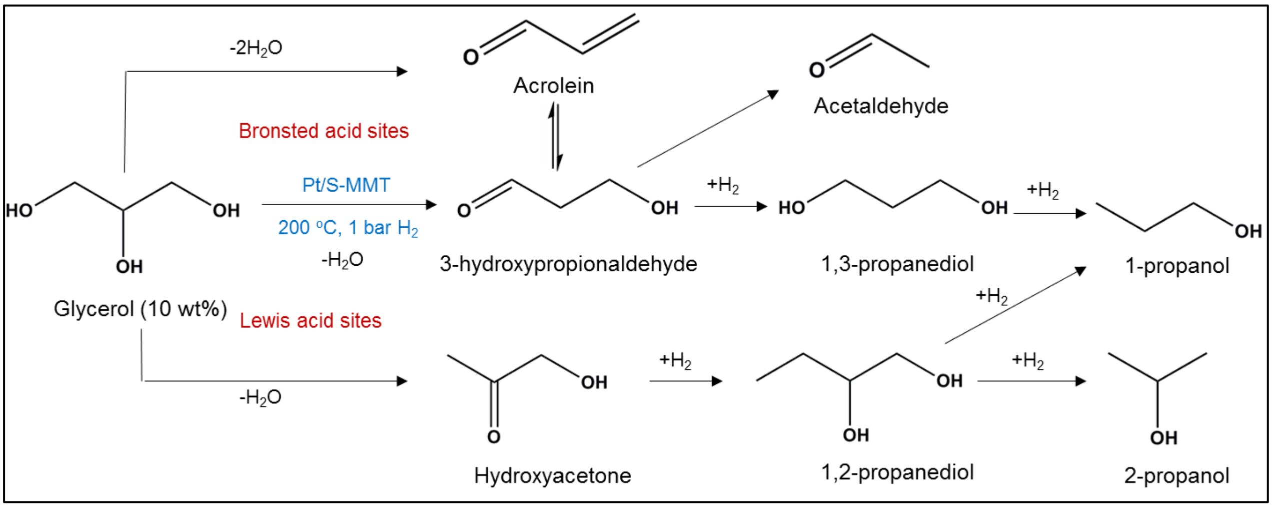

Figure S7. Vapour phase hydrogenolysis of glycerol over Pt/S-MMT catalyst.

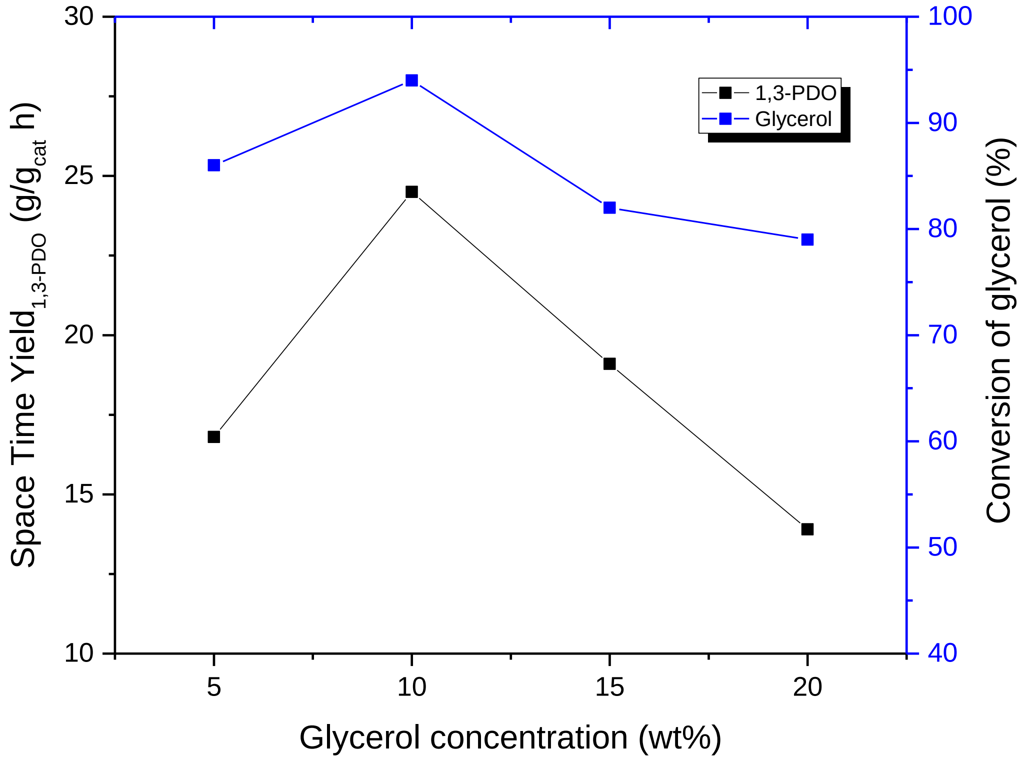

Figure S8. Space Time Yield (STY) of 1,3-PDO at different glycerol concentrations over 2Pt/S-MMT catalyst.

Reaction conditions: Gly conc : 5-20 wt%; 0.5 g of catalyst; reaction temperature of 200 ^{o}C, 1 bar H_2, H_2 flow rate: 70 mL min^{-1}.

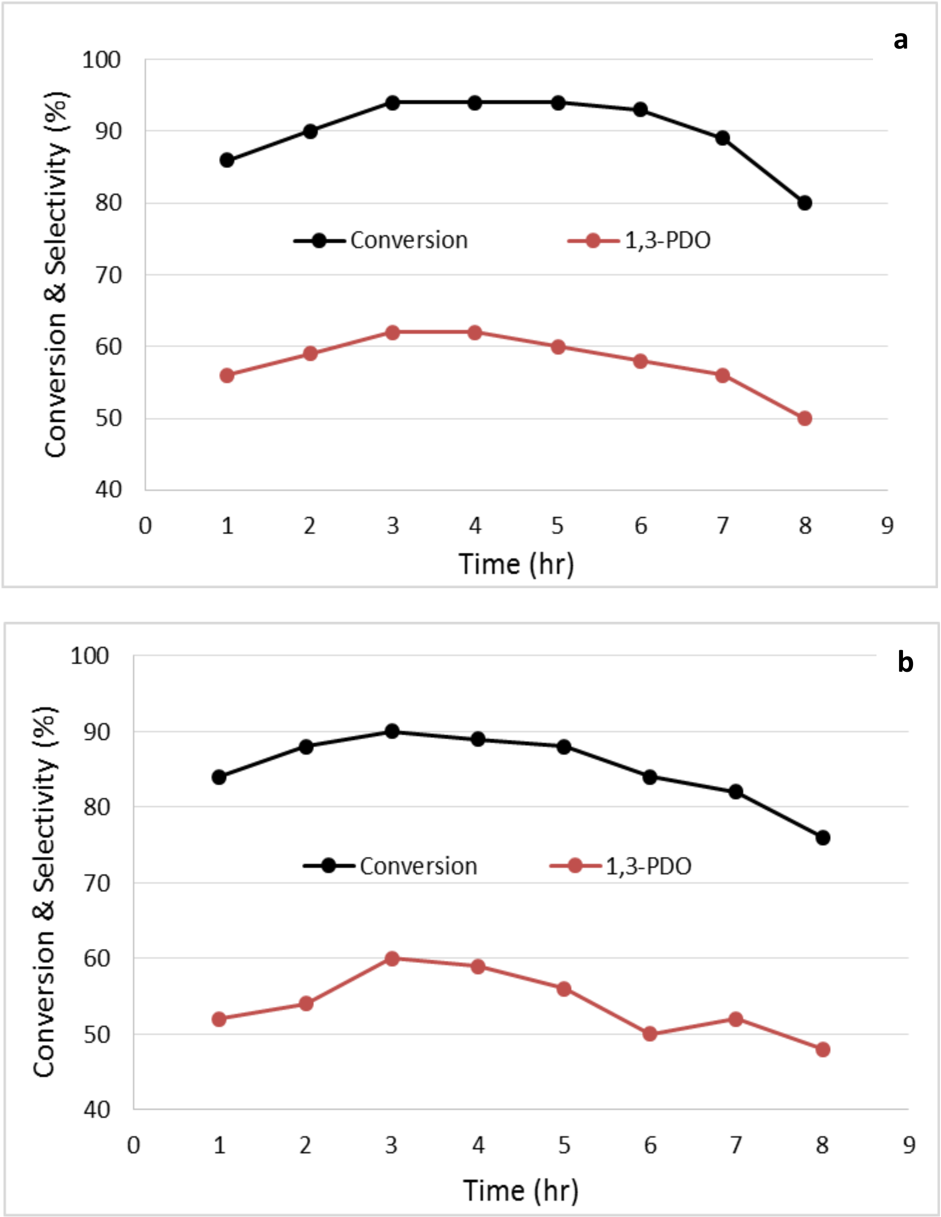

Figure S9. The deactivation behavior and the reuse of the catalyst 2Pt/S-MMT (a) fresh and (b) regenerated/reactivated catalyst.

Reaction conditions: 10 wt% glycerol aqueous solution; 0.5 g of catalyst; reaction temperature of 200 °C, 1 bar H_2, H_2 flow rate: 70 mL min^{-1}.

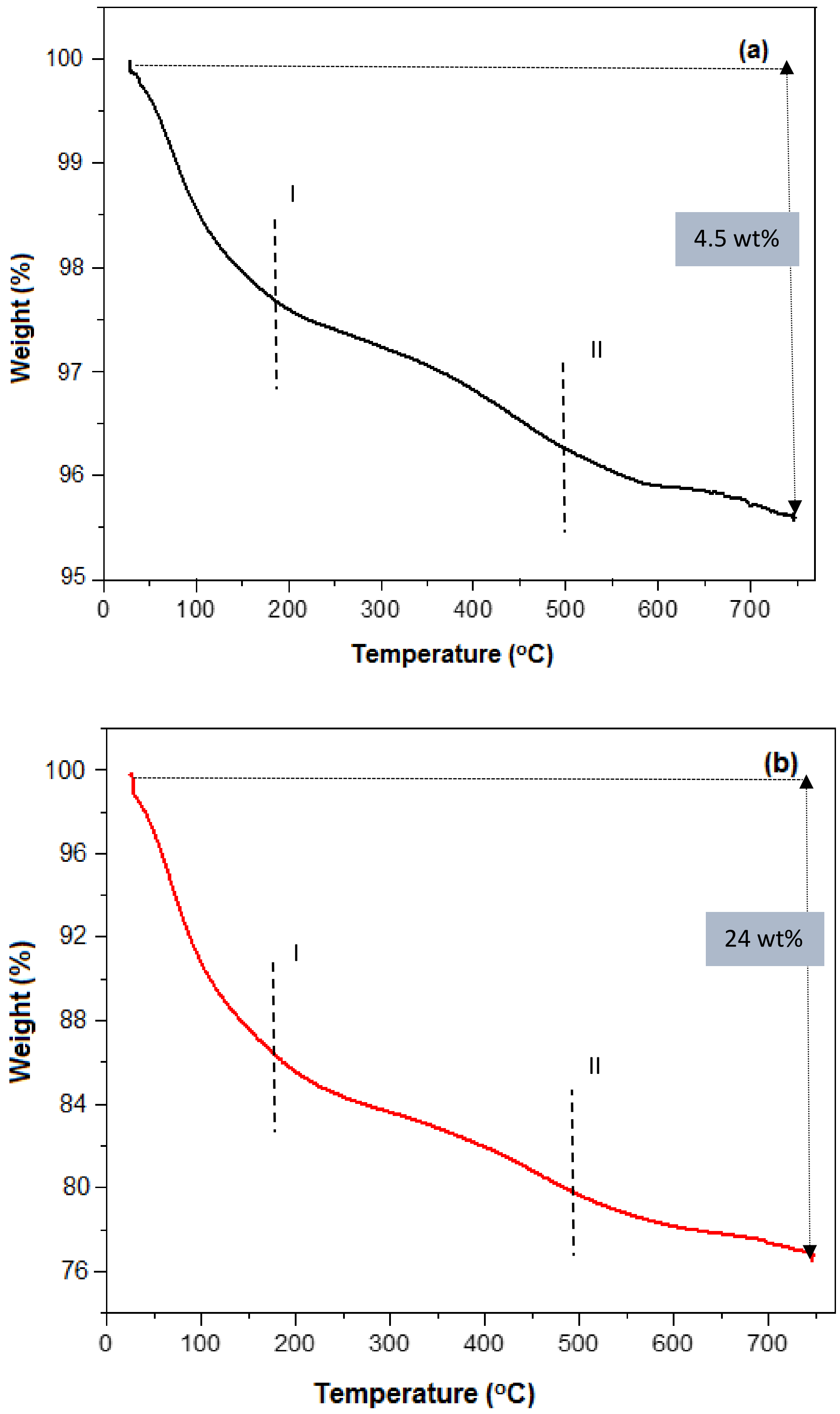

Figure S10. Thermogravimetric analysis (TGA) of the catalyst 2Pt/S-MMT (a) fresh and (b) spent catalyst.

Table S1. Physico-chemical properties of MMT, S-MMT and various Pt/S-MMT catalysts.

Catalyst	S_{BET} (m^2/g)	D_{BJH}(nm)	Vp (cc/g)
MMT	188	5.59	0.26
S-MMT	206	6.36	0.93
0.5Pt/S-MMT	198	5.94	0.79
1Pt/S-MMT	185	5.08	0.67
2Pt/S-MMT	177	4.82	0.52
3Pt/S-MMT	162	3.31	0.39

S_{BET} : BET surface area; D_{BJH} : Average pore diameter; Vp : Total pore volume

Table S2. Results of CO-chemisorption on different Pt/S-MMT catalysts.

Pt (wt%)	Dispersion (%)	CO uptake (μmol/g)	Metal surface area $(m^2/g)_{cat}$	Metal surface area $(m^2/g)_{Pt}$	Particle size[a] (nm)	ICP-AES[b] (wt%)
0.5	72.5	18.6	0.72	145.4	1.92	0.1
1	55.5	28.5	1.11	111.4	2.51	0.6
2	40.1	41.2	1.61	80.5	3.47	1.2
3	30.6	47.2	1.84	61.5	4.55	1.9

[a]Determined from CO uptake values; [b]Metal contents determined from ICP-AES analysis.

Table S3. EDX analysis data of MMT, S-MMT and various Pt/S-MMT catalysts.

**Catalyst**	**Atomic Percentage (%)**					
	**Si**	**Al**	**O**	**C**	**S**	**Pt**
Pure MMT	10.01	3.12	41.79	45.08	--	--
S-MMT	16.17	3.80	61.49	17.04	1.50	--
0.5 Pt/S-MMT	15.84	5.56	62.26	15.63	0.58	0.13
1Pt/S-MMT	16.15	5.63	60.65	16.25	0.55	0.77
2Pt/S-MMT	15.13	5.52	61.21	16.32	0.49	1.33
3Pt/S-MMT	14.93	5.40	61.45	15.50	0.51	2.21

Table S4. Activity of spent catalyst 2Pt/S-MMT.

Catalyst	Conversion (%)	Selectivity of 1,3-PDO	S_{BET} (m^2/g)	Acidity NH_3-TPD (μmol/g)	CHNS analysis (%)			
					C	H	N	S
2Pt/S-MMT (Fresh)	94	62	188	245	0.22	0.41	0.30	0.19
2Pt/S-MMT R (Reactivated after use)	89	59	151	215 (198)	1.61	0.32	0.14	0.14

The value in parenthesis represents the total acidity (μmol/g) of deactivated catalyst measured by NH_3-TPD

Figure Legends

1) Figure S1. Glycerol as a byproduct in biodiesel production process.
2) Figure S2. XRD patterns of MMT, S-MMT and different Pt/S-MMT catalysts.
3) Figure S3. FTIR spectra of MMT, S-MMT and different Pt/S-MMT catalysts.
4) Figure S4. TEM and PSD of PVP capped Pt nanoparticles.
5) Figure S5. NH_3-TPD profile of pure MMT, S-MMT and various Pt/S-MMT catalysts.
6) Figure S6. SEM images and EDX spectra of MMT, S-MMT and 2Pt/S-MMT catalysts.
7) Figure S7. Vapour phase hydrogenolysis of glycerol over Pt/S-MMT catalyst.
8) Figure S8. Space Time Yield (STY) of 1,3-PDO at different glycerol concentrations over 2Pt/S-MMT catalyst.
9) Figure S9. The deactivation behavior and the reuse of the catalyst 2Pt/S-MMT (a) fresh catalyst and (b) regenerated.
10) Figure S10. Thermogravimetric analysis (TGA) of the catalyst 2Pt/S-MMT (a) fresh and (b) spent catalyst.

Tables

1) Table S1. Physico-chemical properties of MMT, S-MMT and various Pt/S-MMT catalysts.
2) Table S2. Results of CO-chemisorption on different Pt/S-MMT catalysts.
3) Table S3. EDX analysis data of MMT, S-MMT and various Pt/S-MMT catalysts.
4) Table S4. Activity of spent catalyst 2Pt/S-MMT.
